# Preparation, Characterization, and Activation of Natural Glassy Carbon Paste Electrodes as New Sensors for Determining the Total Antioxidant Capacity of Plant Extracts

**DOI:** 10.3390/membranes12121193

**Published:** 2022-11-26

**Authors:** Agnieszka Królicka, Anna Szczurkowska, Paweł Mochalski, Grzegorz Malata

**Affiliations:** 1 Department of Building Materials Technology, Faculty of Materials Science and Ceramics, AGH University of Science and Technology, Mickiewicza 30, 30-059 Krakow, Poland; 2 Institute of Chemistry, Jan Kochanowski University of Kielce, 25-406 Kielce, Poland

**Keywords:** antioxidants, polyphenols, epigallocatechin gallate, catechin, carbon paste electrodes, glassy carbon, voltammetry

## Abstract

The continuous search for new sensing materials with high recognition capabilities is necessary to improve existing analytical procedures and to develop new ones. Natural glassy carbon and polydimethylsiloxane were shown to be used for the preparation of carbon paste electrodes to employ them in new, voltammetric, green-chemistry-friendly electroanalytical procedures aimed at evaluating the antioxidant capacity of plant extracts, dietary supplements, and hydrolats. The developed electrodes provided well-shaped and reproducible voltammetric signals (RSD = 1%) of the oxidation of epigallocatechin gallate, the main component of many plants and plant-based formulations with antioxidative activity, in the 1–12.5 µM range (DPV mode, LOD = 0.08 µM). If needed, the performance of new carbon paste electrodes can be further enhanced by the introduction of trivalent rare earth oxides to carbon paste to increase its active surface, facilitate electron transfer, and improve the resolution of recorded signals.

## 1. Introduction

The development of industry in technologically advanced economies is highly dependent on the reliable and fast delivery of results of analytical determinations of hundreds of thousands of substances. As the number of newly synthesized organic and inorganic chemical substances increases exponentially [1], with the number doubling between 2009 and 2015 and reaching more than 250 million substances in 2022 [2], the need for new analytical procedures continues to increase. To meet this task, new sensing materials with high recognition capabilities are necessary. New materials for the construction of sensors can be obtained through advanced materials engineering processes [3] or can be found among naturally occurring materials [4]. Techniques that allow analysis of the composition of samples without subjecting them to additional treatments such as derivatization, separation of the analyte from the matrix, or separation of individual components are of particular interest.

Polyphenols, natural antioxidants, are of interest to many researchers due to their beneficial effects on health and their use in the food industry as natural food preservatives. They are widely used as dietary supplements, additives in pharmaceutical products, and cosmetics [5,6,7]. In the case of testing polyphenols and evaluating their antioxidant properties, it is important that analytical techniques allow the test to be performed without isolating the polyphenols from the matrix, because the desired chemopreventive effect [8] is demonstrated by antioxidants consumed in the form of whole vegetables or fruits and not isolated compounds. The antioxidant activity of food products or nutritional supplements can be evaluated based on spectrophotometric measurement of the Trolox equivalent [8] or by determining total phenolic compounds with the use of Folin–Ciocâlteu reagent [9,10].

Individual polyphenol determinations can be made using both chromatographic [11] and voltammetric techniques, but voltammetry also offers the possibility of determining the total antioxidant capacity of samples. The antioxidant capacity can be assessed on the basis of anodic runs of cyclic voltammograms by measuring peak potential, *E*_p_, and current of the peak, *I*_p_, representing the oxidation process. The more potent antioxidant donates its electrons at a lower potential, which is reflected in the lower value of *E*_p_ and the peak current, *I*_p_, corresponds to the concentration of the antioxidant compound. In the case of multicomponent mixtures of compounds oxidizable at similar potentials, instead of a peak current, the peak area is a more appropriate measure of the antioxidant properties of the examined sample [12,13,14]. To record voltammograms representing anodic reactions, classic GC disk electrodes [15,16] or advanced carbon materials such as carbon nanotubes [16,17,18] can be used. In the case of polyphenol analysis, an important parameter to consider when selecting the electrode type is the regeneration of the electrode surface, as polyphenols easily adsorb on the surface of many materials, including carbon [19]. Adsorbed polyphenols or products of electrode reaction can poison the electrode surface. To avoid this problem, it seems advisable to use carbon electrodes with an easily refreshing surface, i.e., carbon paste electrodes. The fundamentals and a detailed description of the practical aspects of work with carbon paste electrodes were presented in a monumental work published in 2012 [20]. Carbon paste electrodes have only occasionally been used to study polyphenols. Gallic acid was determined in spiked tea samples using graphite/paraffin electrode modified by addition of TiO_2_ [21], by graphite paste electrode modified with carbon nanotubes in wine [22] and together with catechol by graphite/Nujol, carbon microspheres/Nujol, and multiwall carbon nanotubes/Nujol paste electrodes [23]. Rutin was determined in red and white wine samples by Fe_3_O_4_ nanoparticle modified carbon paste electrode [24].

The purpose of the current work was to prepare new carbon paste electrodes with the use of natural glassy carbon, a material not used so far in electroanalysis, and polydimethylsiloxane as a pasting liquid and the assessment of their application in determination of antioxidant properties of plant extracts.

## 2. Materials and Methods

### 2.1. Instrumentation 

The electrochemical study was performed on an Autolab 204 analyzer controlled by Nova 2.1.4 software (Metrohm Autolab, Herisau, Switzerland). Commercial glassy carbon disc electrodes (3 mm in diameter, Mineral, Łomianki-Sadowa, Poland), disc electrodes prepared with laboratory-synthesized glassy carbon and carbon paste electrodes (graphite/paraffin; natural glassy carbon/polydimethylsiloxane) were used as working electrodes. Platinum wire and Ag/AgCl(3 M KCl) were applied as auxiliary and reference electrodes. To record the voltametric curves, the CV or DP (a pulse amplitude of 50 mV) mode was used. The solutions were stirred during the deposition step, which was followed by 5 s of equilibration.

Static contact angles of the electrodes and surface tension were measured using the Attention Theta tensiometer and analyzed using OneAttension 4.1.4 software (Biolin Scientific, Espoo, Finland).

### 2.2. Reagents

Reagents were used as received without further purification. All solutions were prepared using deionized water with a resistivity of 18.2 MΩ (Millipore, Simplicity UV). 

An acetate buffer was prepared by adding 30% NaOH (Suprapur, Merck, Darmstadt, Germany) to a diluted solution of 96% acetic acid (Suprapur, Merck, Darmstadt, Germany) up to the required pH while mixing, using a pH meter. Aqueous solution of catechin and epigallocatechin gallate (Sigma-Aldrich, Darmstadt, Germany) were kept in the refrigerator. 

### 2.3. Electrode Preparation 

#### 2.3.1. Disc Electrodes 

Before use, the disc electrodes were polished using an Al_2_O_3_ suspension (0.3 and 0.05 µm) applied to a polishing cloth and rinsed with water. 

#### 2.3.2. Carbon Paste Electrodes 

The pastes were prepared manually by mixing carbon powder and paste forming liquid in an agate mortar following the procedure described previously [25,26,27]. After homogenizing, the pastes were transferred to a 1 mL syringe barrel. The paste inside the syringe was compressed with a plunger and squeezed into its tip. Electrical contact was provided by a copper wire attached to the plunger seal with a silver tape. After preparation and between experiments, the electrodes were stored immersed in distilled water. It took 2 to 5 days for the freshly prepared paste to stabilize its properties by self-homogenization [25].

Pieces of natural glassy carbon commercialized as a material for water filtration (GC_n_) (Szungite Elite, Myszków, Poland) of approximately 1–2 cm in size were initially crushed in an agate mortar and then ground in a ball mill to a grain size of <63 micrometers. Subsequently, the powdered GC_n_ was further ground in mortar to a grain size <10 micrometer. The 0.35 g portion of GC_n_ was mixed with 0.15 g of pharmaceutical grade polydimethylsiloxane (PDMS) (Almirall, Warsaw, Poland) to produce 0.2 mL of GC_n_ paste. Modified GC_n_ paste electrodes were prepared by substituting 5% carbon by Y_2_O_3_ (Sigma-Aldrich, Darmstadt, Germany), Yb_2_O_3_ (Sigma-Aldrich, Darmstadt, Germany), La_2_O_3_ (Sigma-Aldrich, Darmstadt, Germany), or mixture of Y_2_O_3_ and Yb_2_O_3_ (1:1 mixture). 

The graphite puriss (Fluka, Darmstadt, Germany) and paraffin (Uvasol, Merck, Darmstadt, Germany) were used for the preparation of a classic carbon paste for reference studies. 

### 2.4. Sample Preparation 

Herbs known to have antioxidant properties (*Camellia sinensis*, leaves; *Salvia sclarea* pre-ground leaves), dietary supplement offered as containing green tea polyphenols (tablets), and hydrolat commercialized as a preparation exhibiting antioxidant properties were obtained from local grocery stores. Herbal infusions of *Camellia sinensis* (two brands: green tea 1 and green tea 2, labeled GT1 and GT2) and *Salvia sclarea* (labeled SC) were prepared by brewing for 10 min with 0.15 g of plant material and 100 mL of 95 °C water in a 250 mL beaker covered with watch glass. The herbal samples were neither dried nor further ground, in order to recreate the real conditions of the preparation of herbal infusions. Samples in the form of tablets were ground into powder and then extracted as described above. To remove the insoluble particles, the solution was centrifuged. The solutions obtained by extraction of the dietary supplement were labeled SP. The hydrolat samples were used as received and labeled as HD. 

### 2.5. Voltammetric Procedure 

Electrochemical measurements were performed in a 20 mL vessel at room temperature using 0.1 acetate buffer (pH = 4.5). After the curves representing the background were recorded, aliquots of the examined solutions were added to the buffer to obtain the required concentration. Between experiments, standard solutions of 0.01 M catechin and 0.01 M epigallocatechin gallate were kept at 4 °C in the dark.

### 2.6. Measurement of Contact Angle and Suface Tension

A 3 µL droplet of deionized water was deposited using the sessile drop technique on glassy carbon discs or carbon paste on alumina plates. The surface tension of paraffin and PDMS was measured by the pendant drop approach.

### 2.7. Determinationof Total Phenolic in Herbal Extracts

The total phenolic content of the extracts was determined using the Folin–Ciocâlteu reagent [10]. The 0.5 mL portion of freshly prepared water extract of the tested samples or hydrolat was mixed with 0.5 mL of 70% acetone. Then, 5 mL of 2% Na_2_CO_3_ in 0.1 M NaOH was added. After 5 min, 0.5 mL of Folin–Ciocâlteu reagent was introduced. The absorbance at 760 nm was measured after 120 min, time necessary for color development.

## 3. Results and Discussion

### 3.1. Natural and Synthetic Glassy Carbon

**Figure 1:**
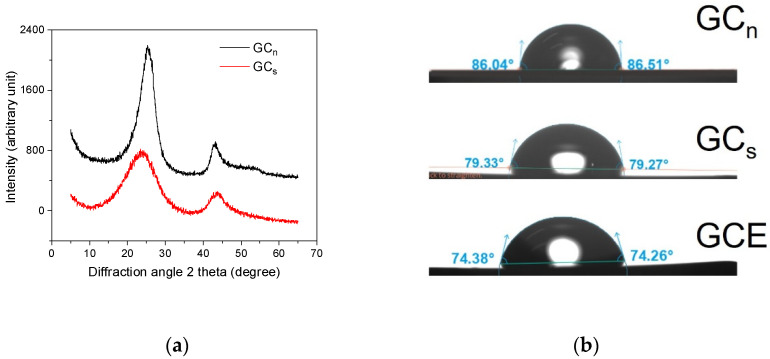
(**a**) X-ray diffraction (XRD) pattern of natural, GC_n_, and synthetic glassy carbon, GC_s_. (**b**) Photographs illustrating the contact angle for the 3 µL drop of water deposited on the surfaces of GC_n_, GC_s_, and commercial glassy carbon electrode, GCE.

Natural glassy carbon in the form of unworked pieces resembles other naturally occurring amorphic volcanic rock, namely obsidian, the volcanic glass commonly used in jewelry making. GC_n_ exhibits a metallic sheen, high hardness, and brittleness. When crushed, it cracks with sharp edges similar to those of other glassy materials. Its unique feature is its ability to conduct electricity, which allows GC_n_ to be considered a potentially useful material for the fabrication of voltammetric electrodes that replace glassy carbon produced by thermal pyrolysis of carbonaceous resins [28]. Figure 1 shows the XRD patterns of a natural glassy carbon sample, GC_n_, (Figure 1, curve a) and a laboratory synthesized glassy carbon sample, GC_s_, (Figure 1, curve b). The presence of two broad and diffuse maxima in both patterns confirms the presence of a fully glassy phase in both natural and synthesized samples of GC [29]. Both GC_n_ and GC_s_ contain 99.7 ± 0.2% carbon as determined on the basis of the loss on ignition (1050 °C, 45 min) and the results of the XRF analysis. 

Another parameter that was used for the characterization of GC_n_ is the contact angle. The contact angle for GC_n_ is slightly higher than that measured for both laboratory synthesized GC_s_ and glassy carbon used for the construction of commercial glassy carbon electrodes, GCE [30], but the obtained value of to 86 ± 1° is close to the 80°, a value typical for glassy carbon (Figure 1b) [28].

### 3.2. Natural Glassy Carbon Paste Electrode 

**Figure 2:**
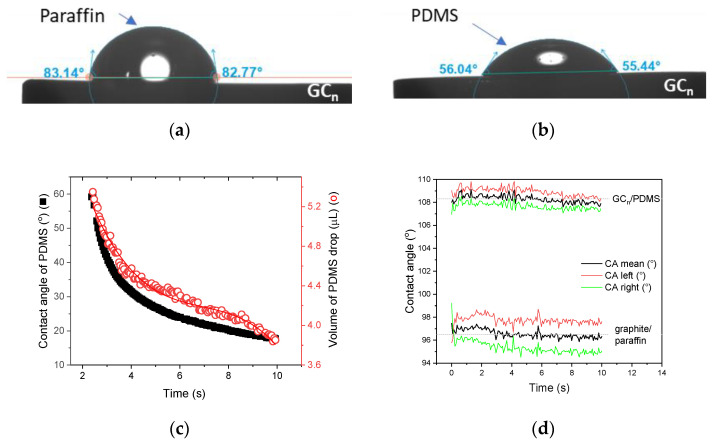
Photographs illustrating the contact angle for (**a**) paraffin drop and (**b**) PDMS drop deposited onto a GC_s_ disc. (**c**) Change in an average contact angle of the PDMS droplet deposited onto the GC_n_ surface and the volume of the PDMS droplet over time. (**d**) Changes in left, right, and mean contact angles of water droplets deposited onto graphite/paraffin and GC_n_/PDMS paste.

Machining of glassy carbon into predefined geometries, for example discs used for construction of classic voltammetric electrodes, is difficult because of the hardness and brittleness of the material. To overcome this kind of difficulty, the synthetic glassy carbon is cast in the form of cylinders, which are then sliced into disks of appropriate thickness to manufacture glassy carbon electrodes, GCE. In the case of natural glassy carbon, another option seems to be more useful, that is the production of carbon paste electrodes, CPEs. To produce the carbon paste, powdered carbon-containing material and paste-forming liquid are needed. As pasting liquids paraffin (γ = 24.1 ± 0.6 mN/m) and PDMS (γ = 24.1 ± 0.6 mN/m) were chosen and two types of carbon paste electrodes were prepared, that is (i) the classic carbon paste electrode, made by mixing graphite of spectral grade with paraffin oil, and (ii) the new containing powdered GC_n_ and PDMS. The first difference in the properties of the prepared pastes can already be observed during their preparation. The workability of the GC_n_/PDMS paste was significantly better compared to that of the graphite/paraffin paste. The better rheological properties of the PDMS paste resulted from the low surface tension of silicone oil, the good wettability of the GC by PDMS (contact angle of PDMS = 56 ± 2° vs. contact angle of paraffin 83 ± 3° Figure 2a,b), and its remarkable ability to spread quickly on the glassy carbon, a property manifested as a rapid change in contact angle and a rapidly decreasing volume of PDMS droplets on the surface of the GC surface (Figure 2c, Video S1). 

During the next stage of the investigations, the carbon pastes were characterized by means of contact angle measurements using water as the probe liquid. By comparing the values of the contact angles on the right and left, it was possible to assess the homogeneity of the surface examined at a distance equal to the diameter of the drop (e.g., for a drop of 3 µL drop, the distance between the measurement points on the left and on the right side of the drop is 2.6 mm). Figure 2d shows the results of the contact angle tested for 10 s after deposition of the water drop on the paste surface. As can be seen from the studies, the GC_n_/PDMS paste is highly hydrophobic (average contact angle of 108.3 ± 0.5) and homogeneous, as evidenced by a small difference between the left and right contact angles—1.2 ± 0.1° on average (Figure 2d). The surface of graphite/paraffin paste was also hydrophobic, but the contact angle was much lower—96.5 ± 0.4° on average and less homogenic, as evidenced by the greater difference between the contact angles left and right than in the case of GC_n_/PDMS. 

**Figure 3:**
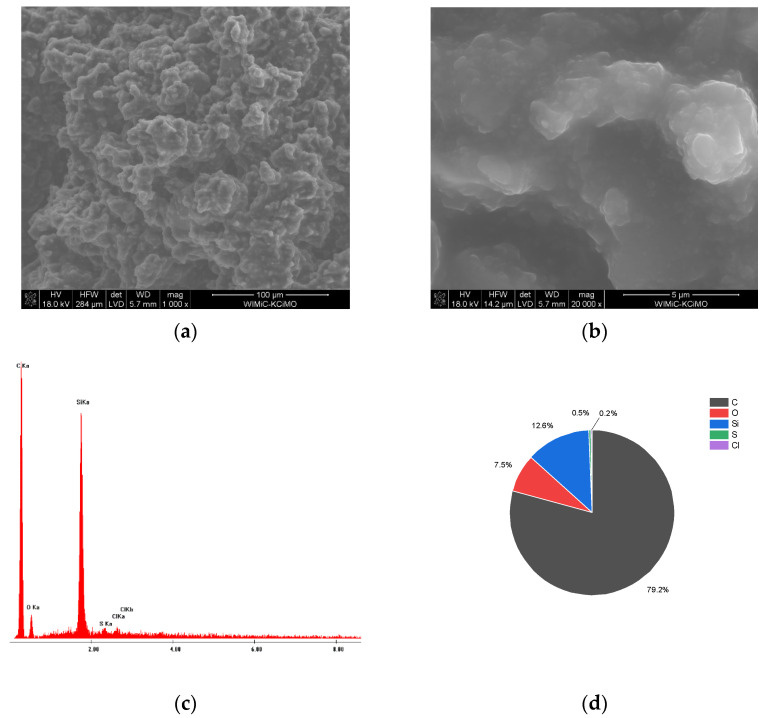
(**a**,**b**) SEM micrographs of GC_n_/PDMS paste taken at different magnifications: (**a**) 1000× *g*, (**b**) 20,000× *g*. (**c**) EDX spectrum of GC_n_ paste, and (**d**) percentage (m/m) composition of GC_n_ paste calculated from EDX data.

Figure 3a,b show the SEM images of the GC_n_/PDMS carbon paste. The studies revealed a porous three-dimensional structure of the examined paste that would be beneficial for the accumulation by adsorption of the targeted compounds. At a magnification of 20,000× *g*, it became apparent (Figure 3b) that fine grains of GC_n_ with a size of approximately 0.2 micrometers have a large share in the paste composition. The large proportion of small grain sizes and lack of agglomeration have a positive effect on the surface development of the electrode material. The EDS analysis of the GC_n_/PDMS paste indicated, in addition to the main elements C, Si, and O, a trace amount of S and Cl (Figure 3c,d). 

### 3.3. Voltammetric Performance of GC_n_/PDMS Electrode

**Figure 4:**
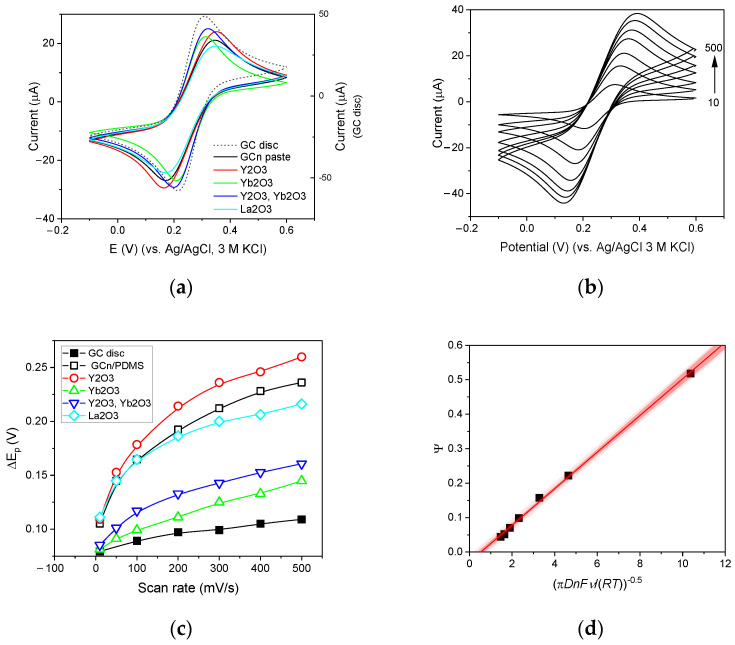
(**a**) Cyclic voltammograms of 0.005 M K_3_[Fe(CN)_6_] recorded in 1 M Na_2_SO_4_ by a GC disc electrode, an unmodified paste electrode GC_n_/PDMS, and GC_n_/PDMS paste electrodes modified with oxide addition at a scan rate of v = 100 mV/s. (**b**) Cyclic voltammograms of 0.5 mM 0.005 M K_3_[Fe(CN)_6_] recorded in 1 M Na_2_SO_4_ using a GC_n_ / PDMS paste electrode at scan rates of 10, 50, 100, 200, 300, 400 and 500 mV/s. (**c**) The peak-to-peak separation Δ*E*_p_ measured for K_3_[Fe(CN)_6_] voltammograms recorded by the GC disc electrode, the unmodified GC_n_/PDMS paste electrode and GC_n_/PDMS paste electrodes modified by oxide addition. (**d**) Plot of *Ψ* vs. [πDnFv/RT]^−0.5^ was prepared using data provided in Figure 4b K_3_[Fe(CN)_6_].

Using the [Fe(CN)_6_]^3−^/^4−^ redox probe in 1M Na_2_SO_4_, the electroactive surface area of the paste electrodes was estimated. The potential was scanned linearly with time within a potential window of −0.1 to 0.6 V with scan rates in the range of 0.01 to 0.5 V/s. According to the calculations, the active surface of GC and GC_n_/PDMS constitutes only 61% or 45% of their geometric area (Table 1). Both the anodic and cathodic peaks obtained on the GC disc and the GC_n_/PDMS paste electrodes were fully developed (Figure 4a), the formal potential E_1/2_ and the value of the i_pa_/i_pc_ ratio had a similar value for both electrodes (Table 1). The peak-to-peak separation ΔE_p_ measured for GC_n_/PDMS electrode was 1.8 times higher than that obtained for the classic disc GC electrode (at v = 0.1 V/s) and quickly increased with increasing polarization rate (Figure 4b,c), which is typical for quasi-reversible systems. To assess the properties of the GC_n_/PDMS electrode, the heterogeneous electron transfer rate constant, *k*^0^, was determined using the Lavagnini approach [31,32]. For this purpose, an empirical relationship between the dimensionless kinetic parameter *Ψ* and Δ*E*_p_xn was used. The value of k^0^ was calculated as the slope of *Ψ* = k^0^[πDnFv/RT]^−0.5^, where D denotes the diffusion coefficient of the electroactive species, *n* is the number of electrons transferred in the electrochemical reaction, F is the constant of the Faraday constant, R is the molar gas and T is the absolute temperature. The value of *Ψ* was calculated using Equation (1), where *X* indicated ΔE_p_xn expressed in mV.

(1) Ψ=(−0.6288+0.0021 X)/(1−0.017 X)

In Figure 4d, the *Ψ* values were plotted against [πDnFv/RT]^−0.5^ revealing the linear dependence (r^2^ = 0.9985) with a slope of 5.31 ± 0.08 × 10^−5^ cm/s. Considering the fact that k^0^ values reported for GC disc electrodes are usually higher than that obtained for GC_n_/PDMS, an attempt to modify the GC_n_/PDMS paste composition to increase the rate of electron transfer was made. 

### 3.4. Modification GC_n_/PDMS Paste by Y_2_O_3_, Yb_2_O_3_, La_2_O_3_ Oxides

**Figure 5:**
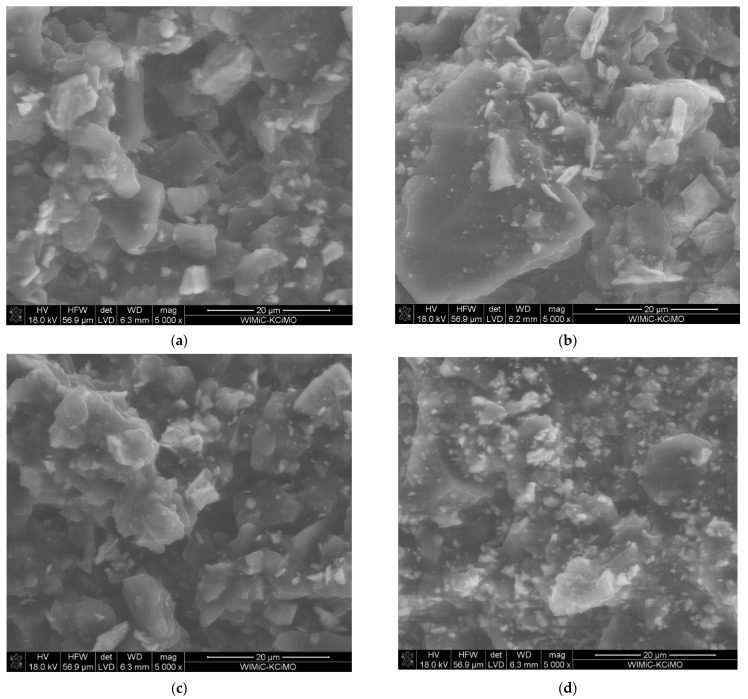
SEM micrographs of oxide modified GC_n_/PDMS paste electrodes taken at 5000× *g* magnification (**a**) Y_2_O_3_, (**b**) Yb_2_O_3_, (**c**) Y_2_O_3_, Yb_2_O_3_, and (**d**) La_2_O_3_.

Three oxides, Y_2_O_3_, Yb_2_O_3_, and La_2_O_3_ and a 1:1 mixture of Y_2_O_3_ and Yb_2_O_3_ were selected to modify the GC_n_/PDMS paste by replacing 5% of GC_n_ with the corresponding oxide. The morphology of the modified electrodes was examined by SEM (Figure 5). The images indicate that modified pastes are homogenous with evenly distributed modifier particles appearing as light gray objects, rectangular or isometric in shape. The addition of a modifier did not cause significant changes in the wettability of its surface, but had an essential influence on the active surface of the electrode (Table 1). The addition of each of the modifiers increased the active surface of the electrodes; however, the effect was not identical. The increase in area was greatest for the addition of Yb_2_O_3_ and the mixture of Y_2_O_3_, Yb_2_O_3_ (Table 1). When considering the separation of the anodic and cathodic peak, a significant decrease of Δ*E*_p_ was achieved for Yb_2_O_3_ and Yb_2_O_3_, Y_2_O_3_, while the addition of Y_2_O_3_ caused the separation peak to increase. The effect of La_2_O_3_ on Δ*E*_p_ was perceptible for scan rates greater than 200 mV/s. The experiments performed for [Fe(CN_6_)]^3−/4−^ in 0.1 M KNO_3_ showed the same trend of Δ*E*_p_ for unmodified GC_n_/PDMS and all oxide modified electrodes (not shown). The ability of modified pastes to transfer electrons can be listed according to the calculated values of the heterogeneous electron transfer rate constants, *k*^0^, in the following way: GC_n_/PDMS/Yb_2_O_3_ ≈ GC_n_/PDMS/Y_2_O_3_/Yb_2_O_3_ >> GC_n_/PDMS > GC_n_/PDMS/Y_2_O_3_ GC_n_/PDMS/La_2_O_3_ (Table S1). To verify the usefulness range of modified electrodes and the benefits of modification, it is necessary to assess their performance while testing real samples.

### 3.5. Analytical Performance of GC_n_/PDMS Electrode

**Figure 6:**
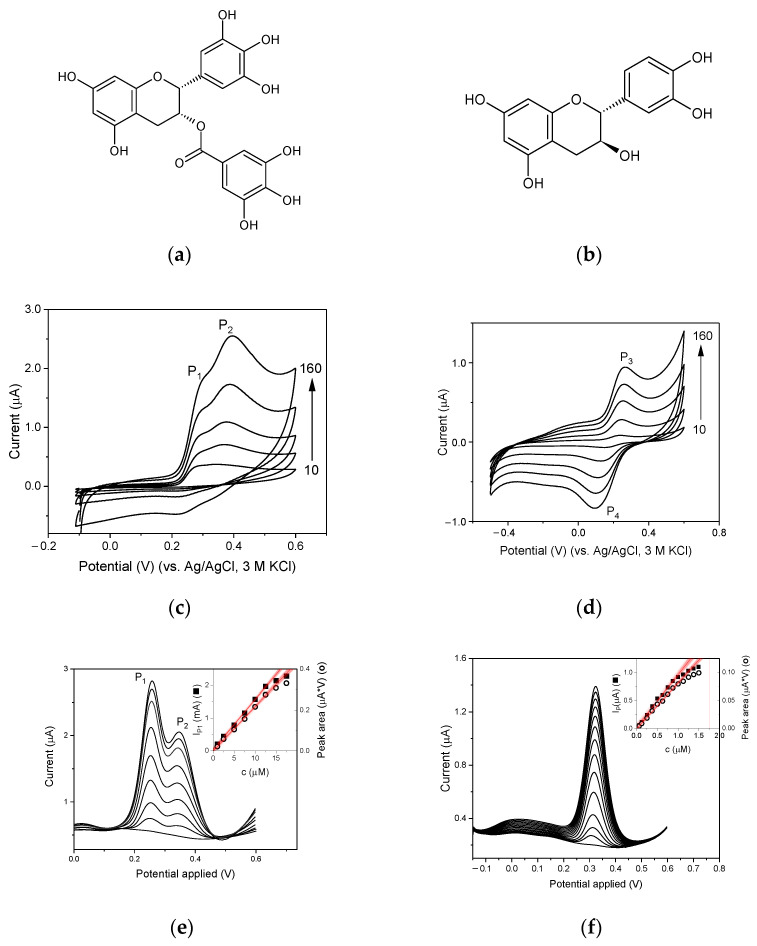
Chemical structures of (**a**) epigallocatechin gallate and (**b**) catechin. Cyclic voltammograms of GC_n_ paste electrode recorded in (**c**) 0.5 mM catechin and (**d**) 0.5 mM epigallocatechin gallate in 0.1 acetate buffer (pH = 4.5) at different scan rates of 10, 40, 80, 120, and 160 mV/s. DP voltammograms recorded using GC_n_/PDMS in 0.1 M acetate buffer containing (**e**) 1–17.5 µM of epigallocatechin gallate and (**f**) 0.06–1.50 µM of catechin. The insets show corresponding calibration plots for the peak current (full square) and the peak area (open circles). Instrumental parameters: accumulation potential, *E*_acc_ = −0.1 V (epigallocatechin gallate), or *E*_acc_ = −0.3 V (catechin), accumulation time, *t*_acc_ = 10 s (epigallocatechin gallate) or *t*_acc_ = 5 s (catechin), modulation amplitude, Δ*E* = 50 mV, potential step, *E*_s_ = 3 mV.

In order to assess the usefulness of the new electrode materials, an attempt was made to use them in the study of solutions of catechins, compounds that naturally occur in many plants and are responsible for their antioxidant properties. As test compounds, two catechins, catechin (Figure 6a) and epigallocatechin gallate (Figure 6b), were selected. The voltammograms obtained recorded with the GC_n_/PDMS paste electrode are shown in Figure 6c,d. Both compounds produced well-shaped oxidation peaks that originated from the processes shown in Figure S1a,b [17,18] (Supplementary Materials), with a very apparent influence of the scan rate on peak current and signal resolution. When plotting the peak current as a function of the scan rate, a linear plot was obtained with the linear regression equation, *I*_p_ = (17 ± 1) *v* + (1 ± 0.1), r^2^ = 0.9952, for P_2_ of epigallocatechin gallate (Figure 6c) and *I*_p_ = (4.1 ± 0.2) *v* + (−0.01 ± 0.02), r^2^ = 0.9940 for P_3_ of catechin, where *I*_p_ is expressed as µA and v in V/s, indicating that the oxidation of both compounds is under adsorption control.

The applicability of the developed electrodes for the determination of epigallocatechin gallate, the main component of tea extracts, was assessed by recording stripping voltammograms in differential pulse mode in 0.1 M of acetate buffer with a pH of 4.5 after accumulation performed at −0.1 V for 10 s. DPV curves were recorded for solutions containing 1–17.5 µM of epigallocatechin gallate. They were well developed throughout the concentration range (Figure 6e). The peak current P_1_ and the area under the peaks P_1_ and P_2_, A_P1,P2_, measured as shown in Figure S2, have a linear relationship with the concentration of epigallocatechin gallate in the range of 1 µM to 12.5 µM (*I*_P1_ = (1.53 ± 0.02) *c* + (0.39 ± 0.17), r^2^ = 0.9990, LOD = 0.08 µM; *A*_P1,P2_ = (2.17±0.05) *c* + (−1 ± 3); r^2^ = 0.9983), LOD = 0.35 µM). The repeatability for 2.5 µM of epigallocatechin gallate and 9 measurements was equal to 1% or 3.8% when the peak current P_1_ or peak area A_P1,P2_ was considered. In the case of catechin, the solution containing 0.06–1.50 µM of catechin was tested. Both the peak current and the peak area of catechin oxidation depended linearly on the concentration as shown in Figure 6f in the range of 0.06 to 0.96 µM (*I*_p_ = (0.94 ± 0.03)*c* + (0.02 ± 0.02), r^2^ = 0.9927, LOD = 0.019 µM; *I*_p_ = (0.082 ± 0.002)*c* + (−0.001 ± 0.001), r^2^ = 0.9965, LOD = 0.025 µM). The repeatability for 0.5 µM of catechin and 9 measurements was equal to 4.5% or 6.2% when the peak current or peak area were considered.

The electrode-to-electrode reproducibility did not exceed 7% measured for peak current. A larger spread of the measured peak currents and less favorable electrode-to-electrode reproducibility are expected for electrodes prepared using different batches of raw GC_n_, because natural materials are always distinguished by a certain composition diversity, depending on the properties of mineral deposits. 

**Figure 7:**
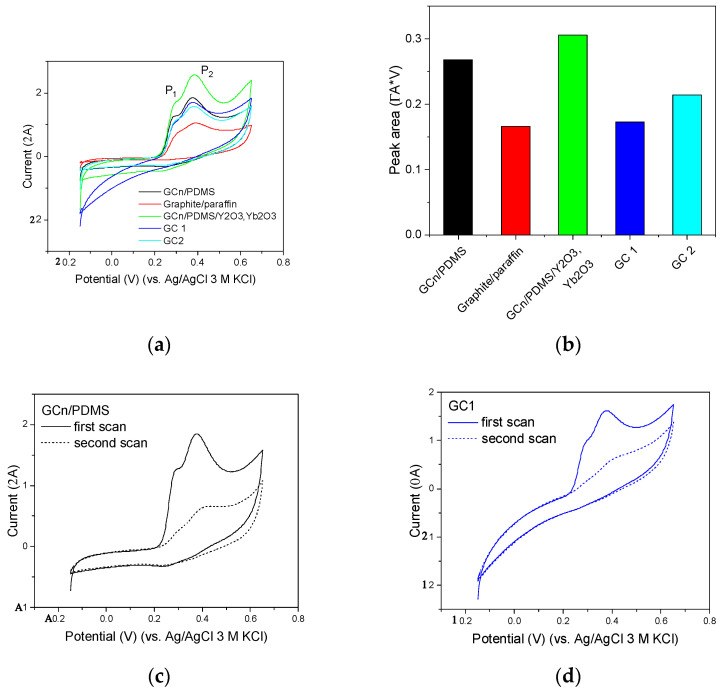
(**a**) Cyclic voltammograms recorded in 5 µM solution of epigallocatechin using paste (GC_n_/PDMS, graphite/paraffin, GC_n_/PDMS modified by Y_2_O_3_, Yb_2_O_3_) and glassy carbon disk (2 mm in diameter, two batches: GC 1 and GC 2) electrodes. (**b**) The area of peaks P_1_ and P_2_ shown in Figure 7a. (**c**,**d**) Two subsequent cyclic voltammograms recorded in 5 µM solution of epigallocatechin gallate using (**c**) GC_n_/PDMS and (**d**) GC 1 electrodes. Supporting electrolyte: 0.1 M acetate buffer, pH = 4.5. Scan rate 50 mV/s.

The performance of GC_n_/PDMS paste electrodes for polyphenols determination can be evaluated on the basis of comparison of epigallocatechin gallate voltammograms recorded using different carbon electrodes, as shown in Figure 7a,b. Epigallocatechin signals recorded using the GC_n_/PDMS electrode reveal two clearly defined peaks, while those recorded with the other electrodes are less developed. For the graphite/paraffin electrode, the peak P_1_ is highly distorted, limited to a shoulder of a more pronounced peak P_2_. The intensity of the signal measured as the area under peaks P_1_ and P_2_ is also more beneficial in the case of GC_n_/PDMS. In addition, it can be further increased by modification of the composition of the carbon paste by adding trivalent oxides of rare earth elements (Figure 7b). The use of paste electrodes helps to address the main issue related to determination of catechins, namely interferences originating from strong adsorption of the catechins and poisoning of the electrode surface by products of electrode processes. The scale of the problem can be assessed by comparing the intensity of epigallocatechin signals obtained in the first and second scans of CV curves (Figure 7c,d). As shown, the signals observed on the second scan are six (GC_n_/PDMS electrode) or eight (GC 1 electrode) times smaller than those observed on the first scan. Due to the blockage of the active sites of the working electrode, the use of electrodes with an easily refreshable surface is recommended. As it is much easier to refresh the surface of paste electrodes than of disc electrodes, this is a strong argument in favor of the application of developed GC_n_/PDMS-based electrodes for polyphenol studies.

### 3.6. Application of GC_n_/PDMS Electrodes for the Determination of Antioxidant Capacity of Herbal Extracts

**Figure 8:**
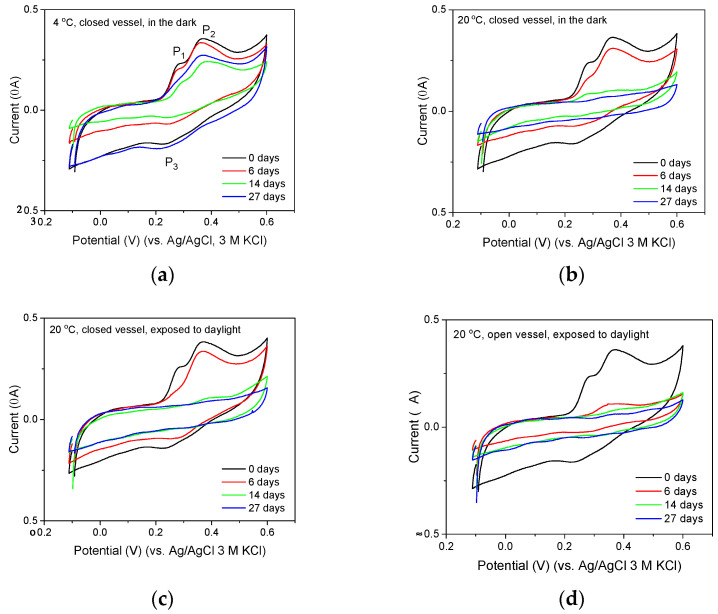
Cyclic voltammograms recorded in 20 times diluted green tea extracts (GT1) stored at (**a**) 4 °C or (**b**–**d**) 20 °C, in (**a**–**c**) sealed or (**d**) opened vessels, (**c**,**d**) exposed to daylight, or (**a**,**b**) kept in the dark. CVs recorded using GC_n_/PDMS electrode in 0.1 acetate buffer (pH = 4.5) with a scan rate of 50 mV/s.

The next stage of the research was the application of GC_n_/PDMS electrodes for the examination of catechins contained in herbal extracts. Opening experiments were carried out in solutions obtained during a 10-min extraction of the *Camellia sinensis* (green tea, GT1) sample with water at 95 °C, using 0.15 g of tea leaves and 100 mL of water. The voltammograms of samples diluted 20 times in 0.1 acetate buffer showed two well-shaped oxidation peaks, P_1_ and P_2_, and a poorly developed reduction peak, P_3_, (Figure 8, black voltammograms) that indicated the presence of epigallocatechin gallate in the sample. Subsequent experiments were aimed at determining whether the sensitivity of the electrodes would be sufficient to track changes in catechins concentration during the storage of tea extracts under various conditions. As can be seen from the voltammograms shown in Figure 8, the GC_n_/PMDS electrode allows tracking of catechins concentration changes caused by temperature, exposure to sunlight, and oxygen access. The recorded voltammograms confirm that solutions exposed to oxygen and sunlight lose their antioxidant properties very quickly (Figure 8d). When access to oxygen is limited, changes occur much more slowly. The high sensitivity and capability of GC_n_/PMDS to provide well-developed catechins signals make it possible to observe subtle changes in the composition of examined extracts caused by photochemical reactions. This is indicated by a comparison of the voltammograms recorded after six days of storing the extracts in the dark and in the presence of sunlight. Two peaks P_1_ and P_2_ are visible on the voltammogram recorded in the solution stored in the dark (Figure 8b), while for the curve recorded in the solution exposed to sunlight (Figure 8c) the mentioned peak P_1_ is no longer observed. The disappearance of the P_1_ peak indicates that, as a result of the action of light, the concentration of epigallocatechin in the tested solution becomes smaller and the solution lost its antioxidant capacity.

**Figure 9:**
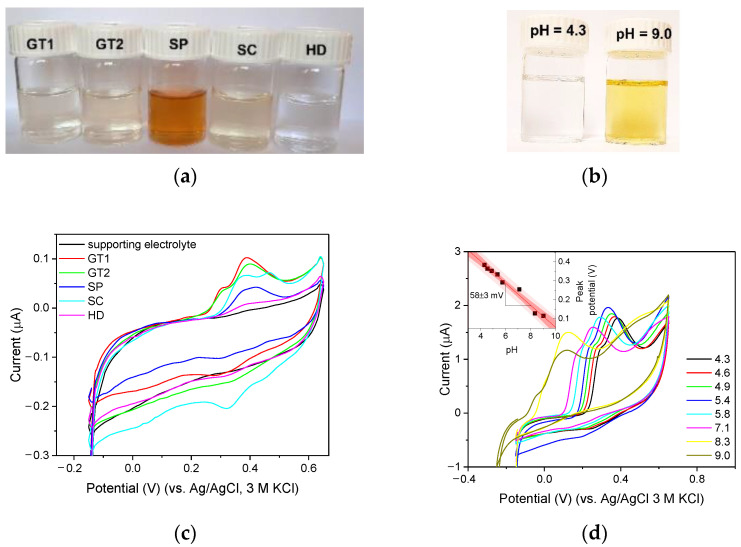
(**a**) Photograph of the solutions before testing: (GT1, GT2) infusion of *Camellia sinensis* (two brands) and (SC) *Salvia sclarea*, (SP) extract of a dietary supplement and (HD) hydrolat. (**b**) Photograph of the 0.1 mM solution of epigallocatechin gallate with pH 4.3 and 9.0. (**c**) Cyclic voltammograms recorded in 200 times diluted extracts using GC_n_/PDMS carbon paste electrodes in 0.1 acetate buffer (pH = 4.5) at a scan rate of 100 mV/s. (**d**) Cyclic voltammograms recorded in 0.1 mM solution of epigallocatechin gallate in 0.1 acetate buffer of different pH using a GC_n_/PDMS carbon paste electrode at a scan rate of 50 mV/s. Inset: The dependence of epigallocatechin peak potential on pH.

Further experiments expanded the scope of plant-based samples and involved other herbal infusions (*Camellia sinensis*, leaves, green tea, GT2; *Salvia sclarea*, pre-ground leaves, SC), a dietary supplement (in tablet form; according to the manufacturer, ‘containing natural polyphenols present in green tea’, SP), and a plant hydrolat (clear liquid, aqueous layer obtained by hydrodistillation of plant material, supposed to exhibit antioxidant properties according to the manufacturer, HD). A 0.1 mL portion of freshly prepared solution (Figure 9a) was added to 19.9 mL of 0.1 M acetate buffer to assess its antioxidant properties. The voltammograms presented in Figure 9c confirm that herbal infusions show a strong antioxidant effect, as large oxidation signals are observed. In the case of a dietary supplement, the oxidation signals are significantly lower. When the hydrolat solution was considered, the oxidation peak did not differ much from the background, indicating no or minimal antioxidant effect. Detailed information on the physicochemical properties of the samples tested is provided in Table 2. By analyzing the data summarized in Table 2, the reasons for the low antioxidant activity of the dietary supplement can be found. Taking into account the instability of epigallocatechin gallate and catechin in neutral and alkaline solutions [33], the high pH of the extract can be indicated as the factor responsible for the low antioxidant activity of the examined formulation. The increase in pH of epigallocatechin gallate solutions causes their coloration (Figure 9b) and a gradual loss of antioxidant properties when the pH of the examined solution exceeded the value of 5.8 (Figure 9d). Both observations agree well with the data reported earlier [33,34,35,36]. The supplement tested was a multicomponent preparation containing magnesium oxide and calcium pantothenate, components that gave a high pH to the aqueous extracts of SP samples. Table 2 provides information on the content of polyphenols determined by spectrophotometry using Folin–Ciocâlteu reagent [10]. As can be seen, the data on the antioxidant capacity of the tested extracts correlate well with the phenolic content determined spectrophotometrically.

### 3.7. Application of Modified GC_n_/PDMS Electrodes for Determination of Antioxidant Capacity of Herbal Extracts

**Figure 10:**
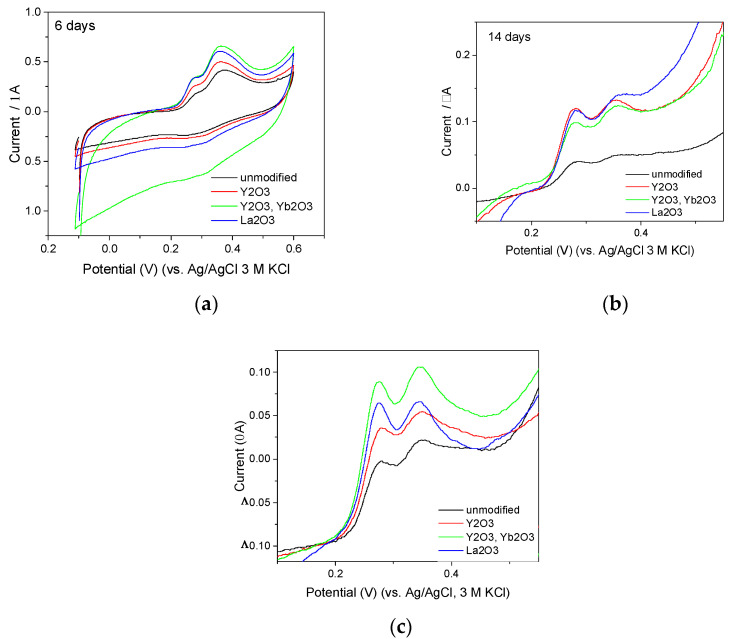
Cyclic voltammograms recorded by unmodified and modified GC_n_/PDMS electrodes in (**a**,**b**) 20 or (**c**) 200 times diluted GT1 extracts, stored in in the dark in closed vessels for (**a**) 6, (**b**) 14, or (**c**) 0 days. (**b**,**c**) Magnified view of the sections of voltammograms presented to highlight the details of the recorded signals. Full voltammograms are shown in Figure S3. Supporting electrolyte: 0.1 M acetate buffer (pH = 4.5). Scan rate: 100 mV/s.

The potential benefits of the application of modified electrodes can be assessed on the basis of the comparison of voltammograms recorded by means of the unmodified and modified GC_n_/PDMS electrodes in solution containing GT1. Undeniably, the modification of the electrodes brought the benefit in the form of better formed and more intense oxidation peaks (Figure 10a); however, the advantages of the modified electrodes are clearly visible only in long-stored solutions, for example, 14 days, when the concentration of the tested compounds is very low (Figure 10b) or in diluted freshly prepared solutions (Figure 10c). 

## 4. Conclusions

The results of the experiments described above indicate that natural glassy carbon can be used as an alternative material for the preparation of paste electrodes. When PDMS is used as a paste liquid, it is beneficial in terms of both rheological characteristics and electrochemical performance (Figure 7a). The high value of the peak-to-peak separation, Δ*E*_p_, and small value of heterogeneous electron transfer rate constant, *k*^0^, obtained for [Fe(CN)_6_]^3−/4−^ voltammograms recorded using the GC_n_/PDMS electrode indicate that electron transfer in the electrode material is slower than in the classic GC electrode. However, the value of *k*^0^ can be doubled by modifying the GC_n_/PDMS paste by adding ytterbium oxide or a mixture of ytterbium and yttrium oxides. Due to modification, the electrochemically active surface of the electrode can also be favorably enlarged. Both modified and unmodified GC_n_/PDMS electrodes can be successfully applied for the electrochemical evaluation of the antioxidant properties of plant extracts. The application of a voltammetric procedure can significantly simplify and shorten the determination of antioxidant activity performed spectrophotometrically through the determination of total phenolic compounds with Folin–Ciocâlteu reagent [10] and/or Trolox equivalent antioxidant capacity [9]. The implementation of a voltammetric procedure with the use of GC_n_/PDMS could be a substantial step toward the dissemination of green analytical procedures by replacing aggressive chemicals (e.g., Folin–Ciocâlteu reagent), minimizing the volume of chemical waste originated from analytical process and substituting materials requiring high-temperature treatment and the use of synthetic precursors.

Upon further examination, the range of possible applications of GC_n_/PDMS electrodes can be extended to

- Stability studies of preparations containing natural antioxidants,
- Research on the photodegrading of antioxidants,
- Evaluation of the influence of individual components on the stability of antioxidant ingredients in the multicomponent formulations,
- Compliance testing of manufacturer’s declaration and actual antioxidant activity of herbal infusions, extracts, and hydrolats.

## Figures and Tables

**Table 1:** Summary of cyclic voltammetric data for disc and paste electrodes. Cyclic voltammograms were recorded in 1M Na_2_SO_4_ containing 0.005 M Fe(CN)_6_^3–^ at a scan rate of 100 mV/s.

Electrode	Modyfier	α	A	E_pa_	E_pc_	Δ*E*_p_	E_1/2_	i_pa_	i_pc_	i_pa_/i_pc_
		(°)	(mm^2^)	(mV)	(mV)	(mV)	(V)	(µA)	(µA)	(−)
Disc, GC *	-	74	4.3	306	217	89	0.262	61	61	1.00
Paste **, GC_n_/PDMS	-	108.3	1.4	340	175	165	0.258	26	27	0.96
Paste, GC_n_/PDMS	Y_2_O_3_	106.6	1.7	346	168	197	0.257	29	30	0.96
Paste, GC_n_/PDMS	Yb_2_O_3_	108.8	2.1	310	211	99	0.261	27	28	0.97
Paste, GC_n_/PDMS	Y_2_O_3_, Yb_2_O_3_	105.7	2.0	318	201	117	0.260	31	31	1.00
Paste, GC_n_/PDMS	La_2_O_3_	104.3	1.5	340	175	165	0.258	23	25	0.94

* GC—commercial disc electrode, 3 mm in diameter, geometric area of 7.1 mm^2^; ** paste electrodes, 2 mm in diameter, geometric area of 3.1 mm^2^.

**Table 2:** Summary of physicochemical and electrochemical parameters of samples.

Sample	Form	Moisture Content (%)	Extract pH	A_P1,P2_ Area (nA *V)	P_1_ (mV)	P_2_ (mV)	TAE * (mg/100 mL)	Antioxidant Effect
GT1	leaves	4.5	5.55	12.5	302	384	7.16	+++
GT2	leaves	4.3	5.35	7.9	306	394	4.70	++
SP	tablet	3.0	7.05	6.1	378	425	- **	+
SC	pre-ground leaves	7.6	6.04	8.2	382	467	5.14	++
HD	hydrolat	-	5.50	1.2	382	-	0.14	negligible

* TAE—equivalent of tannic acid per 100 mL of extract; ** The dietary supplement sample contained soluble silica and non-phenolic compounds [37,38,39] that react with the Folin–Ciocâlteu reagent contributing to overestimation of polyphenol content.

## Data Availability

The data presented in this study are available on request from the corresponding author (A.K.).

## Supplementary Materials

The following supporting information can be downloaded at: https://www.mdpi.com/article/10.3390/membranes12121193/s1, Video S1: PDMS drop on the GC_n_ surface, Table S1: The heterogeneous electron transfer rate constant, k0, obtained for unmodified and modified natural glassy carbon paste electrodes employing the Lavagnini method; Figure S1: Electrode processes assigned to irreversible electrooxidation of (a) epigallocatechin gallate (peaks P_1_ and P_2_), as well as (b) reversible electrooxidation of catechin (peaks P_3_ and P_4_). Figure S2: Illustration of how the peak area (the hatched portion of the graph), A_P1,P2_, was measured for epigallocatechin solutions and in the studies of herbals extracts to determine the antioxidant capacity of the test solutions; Figure S3: Cyclic voltammograms recorded by unmodified and modified GCn/PDMS electrodes in (a,b) 20 or (c) 200 times diluted GT1 extracts, stored in in the dark in closed vessels for (a) 6, (b) 14 or (c) 0 days. Supporting electrolyte: 0.1 M acetate buffer (pH = 4.5). Scan rate: 100 mV/s.
