# Supplementary material for: Preparation, Characterization, and Activation of Natural Glassy Carbon Paste Electrodes as New Sensors for Determining the Total Antioxidant Capacity of Plant Extracts
Source: Membranes (Basel). 2022 Nov 26;12(12):1193. doi: 10.3390/membranes12121193 (PMC9783599; doi:10.3390/membranes12121193)
Supplement: Supplementary file 1 [file membranes-12-01193-s001.zip › membranes-2024604-Supplementary Material.pdf]

Preparation, characterization, and activation of natural glassy carbon paste electrodes as new sensors for determining the total antioxidant capacity of plant extracts

Agnieszka Królicka [1,*], Anna Szczurkowska [1], Paweł Mochalski [2] and Grzegorz Malata [1]

[1] Department of Building Materials Technology, Faculty of Materials Science and Ceramics, AGH University of Science and Technology, Mickiewicza 30, 30-059 Krakow, Poland

[2] Institute of Chemistry, Jan Kochanowski University, 25406 Kielce, Poland

Table S1. The heterogeneous electron transfer rate constant, k^0, obtained for unmodified and modified natural glassy carbon paste electrodes employing the Lavagnini method [1].

Electrode	k^0 (10^{-5} cm/s)
GC_n/PDMS	0.053±0.001
GC_n/PDMS/Y_2O_3	0.050±0.001
GC_n/PDMS/Yb_2O_3	0.11±0.01
GC_n/PDMS/Y_2O_3,Yb_2O_3	0.09±0.01
GC_n/PDMS/La_2O_3	0.042±0.001

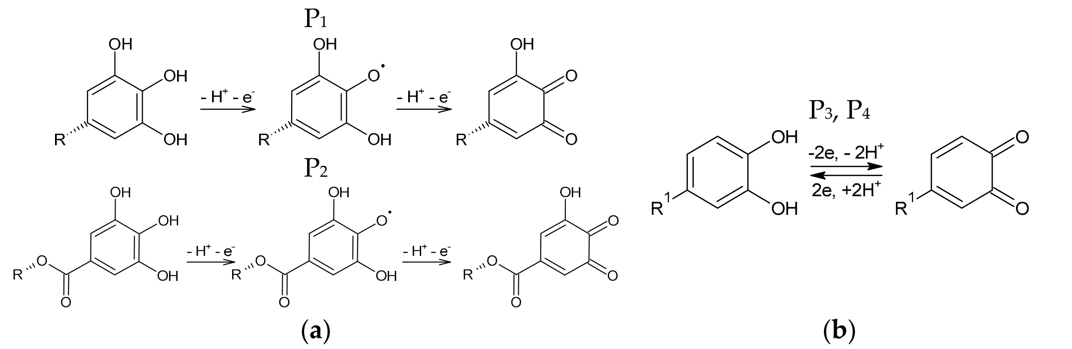

Figure S1. Electrode processes assigned to irreversible electrooxidation of (**a**) epigallocatechin gallate (peaks P_1 and P2), as well as (**b**) reversible electrooxidation of catechin (peaks P_3 and P_4).

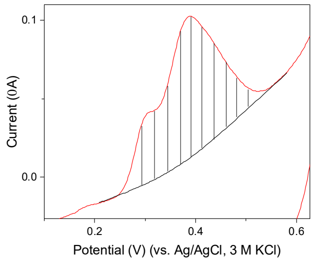

Figure S2. Illustration of how the peak area (the hatched portion of the graph), $A_{P1, P2}$, was measured for epigallocatechin solutions and in the studies of herbals extracts to determine the antioxidant capacity of the test solutions.

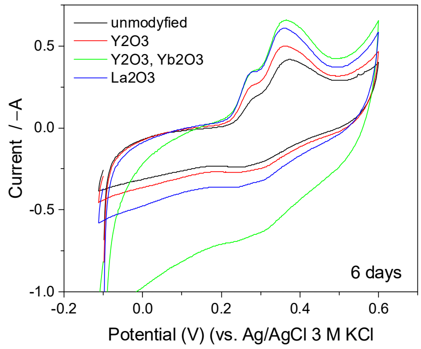

(**a**)

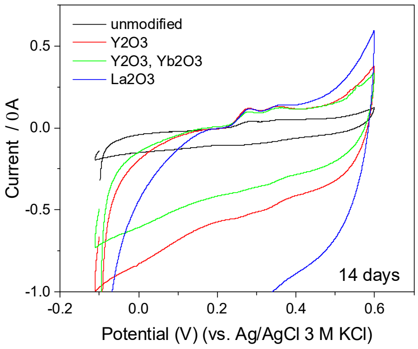

(**b**)

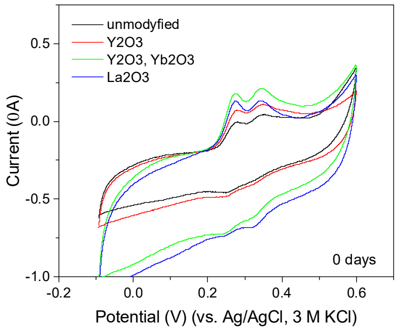

(**c**)

Figure S3. Cyclic voltammograms recorded by unmodified and modified GC_n/PDMS electrodes in (**a** and **b**) 20 or (**c**) 200 times diluted GT1 extracts, stored in in the dark in closed vessels for (**a**) 6, (**b**) 14 or (**c**) 0 days. Supporting electrolyte: 0.1 M acetate buffer (pH = 4.5). Scan rate: 100 mV/s.

1. Lavagnini, I., R. Antiochia, and F. Magno, *An extended method for the practical evaluation of the standard rate constant from cyclic voltammetric data.* Electroanalysis, 2004. **16**(6): p. 505-506.
